# Cost-effectiveness of medical migration for chronic kidney disease: a national cross-sectional study in China

**DOI:** 10.1186/s12913-022-08266-x

**Published:** 2022-07-13

**Authors:** Yumeng Ao, Chao Yang, Pengfei Li, Fulin Wang, Suyuan Peng, Huai-Yu Wang, Jinwei Wang, Ming-Hui Zhao, Luxia Zhang, Ye Yuan, Xuezheng Qin

**Affiliations:** 1 School of Economics, Peking University, Beijing, 100871 China; 2 Renal Division, Department of Medicine, Peking University First Hospital; Peking University Institute of Nephrology, Beijing, 100034 China; 3 Research Units of Diagnosis and Treatment of Immune-Mediated Kidney Diseases, Chinese Academy of Medical Sciences, Beijing, 100034 China; 4 Advanced Institute of Information Technology, Peking University, Hangzhou, 311215 Zhejiang China; 5 Institute of Medical Technology, Peking University Health Science Center, Beijing, 100191 China; 6 Peking University First Hospital, Beijing, 100034 China; 7 National Institute of Health Data Science at Peking University, Beijing, 100191 China; 8 Peking-Tsinghua Center for Life Sciences, Beijing, 100871 China

**Keywords:** Chronic kidney disease, Cost-benefit analysis, Hospital mortality, Length of hospital stay, Medical migration

## Abstract

**Background:**

The phenomenon of medical migration is common in China. Due to the limited capacity and substantial geographical variation in medical practice, patients with chronic kidney disease (CKD) travel more frequently to seek medical care. We aimed to assess the cost-effectiveness of medical migration for CKD patients in China and provide real-world evidence for the allocation of CKD resources.

**Methods:**

Records of patients with CKD between January 2014 and December 2018 were extracted from a large national database. A patient is defined as a medical migrant if she travelled across the provincial border to a non-residential province to be admitted for inpatient care. The propensity score matching method is used to estimate the effect of medical migration on medical expenditure, length of hospital stay, and in-hospital mortality. The cost-effectiveness is evaluated by comparing the estimated cost per life saved with contemporaneous estimates of the value of a statistical life.

**Results:**

Among 4,392,650 hospitalizations with CKD, medical migrants accounted for 4.9% in 2018. Migrant patients were estimated to incur a 26.35% increase in total medical expenditure, experience a 0.24-percentage-points reduction in in-hospital mortality rates, and a 0.49-days reduction in length of hospital stay compared to non-migrant patients. Overall, medical migration among CKD patients incurred an average of 1 million yuan per life saved, which accounted for 20–40% of contemporaneous estimates of the value of a statistical life. Compared with migrant patients with self-payment and commercial insurance, migrant patients with public health insurance (urban basic medical insurance and new rural co-operative medical care) incurred lower cost per life saved. Cost per life saved for CKD patients was similar between female and male, lower among older population, and varied substantially across regions.

**Conclusions:**

The medical care seeking behaviors of CKD patients was prominent and medical resources of kidney care were unevenly allocated across regions. Medical migration led to a reduction in mortality, but was associated with higher medical expenditure. It is imperative to reduce the regional disparity of medical resources and improve the clinical capacity. Our study shows that it is imperative to prioritize resource allocation toward improving kidney health and regional health care planning.

**Supplementary Information:**

The online version contains supplementary material available at 10.1186/s12913-022-08266-x.

## Background

China is a developing country with the largest population with chronic conditions in the world. With the rapid economic development and population aging, the demand for high-quality health care has been growing steadily in China [1]. As medical resources are unevenly allocated across regions and concentrated in more developed areas, an increasing number of patients travel far to seek health care outside their residential areas. According to the National Medical Service and Quality Safety Report, the share of trans-provincial inpatient admissions in tertiary hospitals was 7.16% in 2016. Since public financing of hospitals and administration of public health insurance are both managed at the city or provincial level [2], patients who seek medical care across provincial border have to bear substantially higher medical expenditure and lower insurance reimbursement rates, which leads to increased financial burden to both patients and the society [3]. Despite of these substantial barriers in trans-provincial health care, there is a rising number of patients seeking medical care across provincial borders in China. Therefore, understanding the pattern and cost-effectiveness of trans-provincial medical care utilization may guide a sound policy-making in health care system planning and health insurance design, especially for developing countries with limited medical resources.

The Chinese government launched a healthcare reform in 2009, aimed at providing more affordable and equitable access to health care for all citizens. The reform reinstated the government’s role in financing health care and the provision of public goods [4]. Recently, China has also made efforts to build a referral system and reduce the burden of migrant patients. In 2015, the State Council issued a statement to promote the tiered health-care delivery system to optimize medical resources, including improving both the service quality and capacity of county-level public hospitals [5]. Despite these efforts of the government, the phenomenon of medical migration for major diseases is still prevalent.

In the last decade, chronic kidney disease (CKD) has been recognized as a major public health problem globally [6]. CKD is predicted to rise from the 16th to 5th leading cause of premature death between 2016 and 2040 [7]. A recent nationwide cross-sectional survey in China found that the prevalence of CKD was 10.8% [8]. The growth in CKD population also implies an increasing burden of end-stage kidney disease (ESKD) patients requiring kidney replacement therapy (KRT, either dialysis or kidney transplantation), which consume a disproportionate high percentage of medical expenditure [9–11]. Developed countries like the United States and the Netherlands have established a well-functioning hierarchical medical system [12, 13]. However, many developing countries, including China, still face multiple challenges in provision and utilization of kidney care, including limited clinical capacity, low efficiency in utilization, and substantial geographical variation in medical practice [14, 15].

The disparity in access to kidney care, particularly for KRT, has been widely observed in developing countries, and China is no exception [16, 17]. In China, access to kidney care services is limited because of to a lack of medical resources and substantial regional disparity [15]. Most clinical resources for CKD, particularly those for KRT, are concentrated in large metropolitan cities, and are lacking in smaller cities. Patients often face substantially higher *out-of-pocket* medical expenditure if seeking medical care across provincial borders [18]. Nevertheless, the potential health benefits for such trans-provincial care seeking is understudied in China. Previous literature focuses mostly on the health insurance design and payment reform for migrant patients [19–21]. So far there exists no analysis on the cost-effectiveness of trans-provincial care seeking from a national perspective.

Therefore, understanding the cost-effectiveness of trans-provincial care seeking among CKD patients is critically important not only for improving patient welfare, but also for a sound policy-making in China’s health care system and health insurance system. To simplify the exposition, we hereafter refer to such trans-provincial utilization of medical care as medical migration. Based on a large nationwide database, Hospital Quality Monitoring System (HQMS), we conducted one of the first study to assess the cost-effectiveness of medical migration for CKD patients in China and aimed to provide a stream of crucial empirical evidence for the medical resource allocation and policy development.

## Methods

### Data source

The HQMS database is a mandatory patient-level national database for hospital accreditation, under the authority of the National Health Commission (NHC) of the People’s Republic of China. Details of the HQMS database were described elsewhere [10, 22]. Briefly, the NHC requires that all tertiary hospitals in China submit inpatient discharge records to HQMS in a standardized electronic format on a daily basis. Tertiary hospitals in China are the top tier provider of primary, secondary, and tertiary care to a nationwide patient population. The number of patient visits to tertiary hospitals accounts for more than 50% of total patient visits in China [23]. The HQMS database contains detailed patient-level variables including demographic characteristics, discharge diagnoses, procedures, and medical expenses extracted from the discharge summary known as the “front-page” of hospital medical record. The discharge diagnoses were coded using International Classification of Diseases, Tenth Revision (ICD-10). As of December 2018, HQMS has covered more than 75% of tertiary hospitals in 31 provinces, autonomous regions and municipalities directly under the central government (excluding Hong Kong, Macao, and Taiwan).

### Study population

**Fig. 1:**
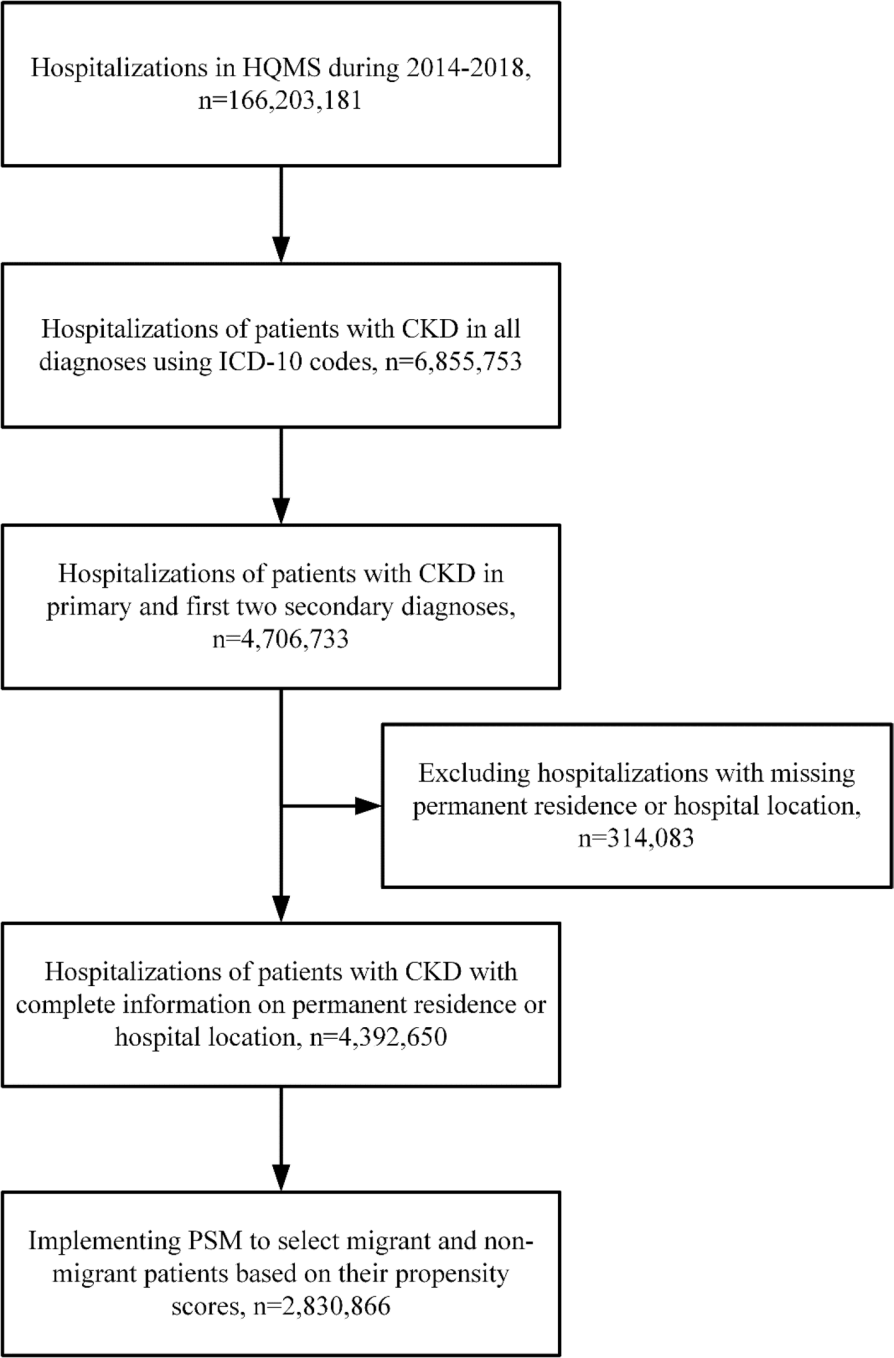
Flow chart of study population selection. Abbreviations: CKD, chronic kidney disease; HQMS, Hospital Quality Monitoring System; ICD-10, International Classification of Diseases, Tenth Revision; PSM, propensity score method

Records of patients with CKD between January 1, 2014 and December 31, 2018 were extracted from the HQMS. Hospitalizations with at least one of the following diagnoses (in both primary diagnosis and the first two secondary diagnoses) were included (relevant ICD-10 coding in Table S1): diabetic kidney disease, glomerulonephritis, hypertensive nephropathy, obstructive nephropathy, chronic tubulointerstitial nephritis, and CKD due to other reasons. Records with missing data of permanent residence or hospital location were excluded. The propensity score matching (PSM) method was implemented to restrict the sample of migrant and non-migrant patients to ensure comparability. Our final study sample included 2,830,866 hospitalizations. The flow chart of study population selection was shown in Fig. 1.

### Definitions of variables

We defined a migrant patient as one who travelled from residential province to another province to be admitted for inpatient care. For such patients, we referred to their residential province as the origin province, and the province of the admitting hospital as the destination province. Comparatively, we defined a non-migrant patient as one who received inpatient care within the residential province. We define a series of control variables including patient’s basic demographic information, insurance type, and common CKD comorbidities. Common comorbidities including cardiovascular disease (CVD), diabetes, and hypertension were identified by ICD-10 codes (in both primary and secondary diagnoses, Table S2). Demographic variables include age and gender; further personal information was excluded for privacy. Health insurance was classified into four main types: urban basic medical insurance (UBMI), new rural co-operative medical care (NRCMC), commercial insurance, and self-paid payment. Key variables on patient treatment outcomes include total medical expenditure, length of hospital stay, and in-hospital mortality.

### Ethical approval

This study was approved by the Ethics Committee of Peking University First Hospital (2020–018). Acquisition of informed consent was exempted. All analyses were completed under the support of the World Health Organization (China-World Health Organization Biennial Collaborative Projects 2018–2019).

### Statistical analysis

Data were reported as proportions (%) for categorical variables, and the mean ± standard deviation (SD) or median (inter-quartile range, IQR) for continuous variables. All analyses were based on the hospitalizations (patient-records), not individuals.

The PSM method was implemented to estimate the causal effect of medical migration on total medical expenditure and treatment outcomes. In our setting, the propensity score was the probability of a patient’s engaging in medical migration conditional on observed characteristics. The logistic regression was used to estimate the propensity score conditional on the patient’s demographics, health insurance, major comorbidities, indicators for residential province, and indicators for the year, month, and day of the week of hospital admission. A nearest neighbor matching algorithm based on the estimated propensity score was then adopted to select a comparison group of non-migrant patients. To further reduce the risk of spurious matches, we imposed a maximum tolerance level (i.e., a caliper of 0.1) of the distance in propensity scores between matched units. We further restricted the matched sample to have a propensity score strictly between 0.1 and 0.9 to ensure the common support.

Based on the matched sample of CKD patients, we estimated the effect of medical migration on patient’s outcomes as the average difference in outcomes between migrant and non-migrant patients. Then we computed the measure of cost per life saved as the ratio of estimated incremental change in total medical expenditure over estimated incremental change in the mortality rate. Finally, we evaluated the cost-effectiveness of medical migration by comparing the cost per life saved with contemporaneous estimates of the value of a statistical life (VSL). The latter measures the economic value that individuals are willing to pay in order to reduce the statistical risk of mortality. VSL is a commonly used metric in benefit-cost analysis to evaluate the efficiency of public policies, especially in public health, occupational safety, and environmental protection [24, 25]. All statistical analyses were performed using STATA 15.0 software (Stata Corp LP, College Station, TX, USA).

## Results

The mean age of hospitalized patients with CKD was 55.0 ± 16.4 years, and 56.4% were men. Compared with non-migrant patients, migrant patients were on average younger, more likely to have self-payment and glomerulonephritis, and less likely to have common comorbidities such as CVD, diabetes, and hypertension. Moreover, migrant hospitalizations were also associated with higher hospitalization costs (9,908 yuan vs. 9,095 yuan) and lower in-hospital mortality rates (0.4% vs. 0.7%) (Table 1). The percentage of medical migration among patients with CKD was 4.5% in 2014 and 4.9% in 2018, respectively, with a slight upward trend.

**Table 1:** Characteristics of hospitalized patients with CKD in China, 2014–2018

	Non-migrant	Migrant	Total
Hospitalizations	4,185,826	206,824	4,392,650
Age (years)	55.3 ± 16.3	47.9 ± 15.7	55.0 ± 16.4
Male (%)	56.3	57.9	56.4
Health insurance (%)			
UBMI	51.4	25.3	50.2
NRCMC	22.6	18.3	22.4
Self-paid payment	12.5	36.7	13.7
Commercial	13.5	19.7	13.7
CKD cause (%)			
Diabetic kidney disease	20.9	11.8	20.5
Hypertensive nephropathy	13.9	11.4	13.7
Glomerulonephritis	20.3	27.2	20.6
Chronic tubulointerstitial nephritis	1.8	1.9	1.8
Obstructive nephropathy	15.7	16.6	15.7
Others	27.5	31.2	27.7
CVD (%)	23.0	13.2	22.5
Diabetes (%)	28.1	17.7	27.6
Hypertension (%)	51.3	42.7	50.9
Cost (yuan)^a^	9,095 (5,272-15,271)	9,908 (5,549-17,149)	9,129 (5,284-15,352)
Length of hospital stay (days)	9 (6–14)	8 (5–14)	9 (6–14)
In-hospital mortality (%)	0.7	0.4	0.7

*Abbreviations CKD* Chronic kidney disease, *CVD* Cardiovascular disease, *NRCMC* New rural co-operative medical care, *UBMI* Urban basic medical insurance

^a^The percentages of missing values for cost was 10.4% for total hospitalizations, 8.7% for migrant hospitalizations, and 10.4% for non-migrant hospitalizations

Compared with the unmatched sample reported in Table 1, characteristics between migrant and non-migrant patients were well-balanced after PSM estimation (Table 2). The matched sample also have good common support in propensity scores (Appendix Figure S1). Migrant patients were estimated to incur 26.35% higher medical expenditure (Table 3), or equivalently, 2,578 yuan (Appendix Table S3), than their non-migrant counterparts. Meanwhile, migrant patients had a 0.24-percentage-points reduction in in-hospital mortality rates and 0.49-days reduction in length of hospital stay compared to non-migrant patients (Table 3). We further tested the sensitivity of these results to different specifications of the PSM algorithm. We restricted the sample according to more stringent matching criteria of one-to-one matching and a caliper of 0.01 (Appendix Table S4). In these specifications, the estimated effects of medical migration were similar to the baseline.

**Table 2:** Test of the balance of characteristics of migrant and non-migrant patients with CKD after PSM estimation

	Mean		T-test	
Variable	Migrant	Non-migrant	T-statistics	***P***-value
**Demographics**				
Male	0.563	0.563	0.190	0.848
Age ≤ 40 y	0.296	0.295	0.840	0.398
40 y < Age ≤ 60 y	0.458	0.459	−0.380	0.702
60 y < Age ≤ 80 y	0.234	0.234	− 0.620	0.537
**Health insurance types**				
UBMI	0.220	0.221	−0.550	0.584
NRCMC	0.288	0.288	−0.020	0.982
Commercial insurance	0.217	0.217	−0.190	0.847
Self-payment	0.268	0.269	−0.690	0.491
**Common comorbidities**				
Hypertension	0.432	0.432	−0.230	0.819
Diabetes	0.173	0.173	−0.210	0.832
CVD	0.123	0.122	1.550	0.120

Propensity score matching method was used to construct the matched sample of the migrant group (trans-provincial migrant patients) and non-migrant group (non-migrant patients). The logistic model was used to estimate the propensity score based on the patient’s demographics, health insurance, major comorbidities, province dummies, and year dummies. The nearest neighbor algorithm was used with a caliper of 0.1. The sample was further restricted to have common support and had scores strictly between 0.1 and 0.9

*Abbreviations*: *CVD* Cardiovascular disease, *PSM* Propensity score matching, *UBMI* Urban basic medical insurance, *NRCMC* New rural co-operative medical care

**Table 3:** Estimated effects of medical migration on expenditure and health outcomes of patients with CKD

	Coefficient	Standard Error	T-statistics	*P*-value	Obs (migrant)	Obs (non-migrant)
**Expenditure (log)**	0.2635	0.0016	166.74	< 0.001	809,379	2,021,487
**Mortality rate**	−0.0024	0.0001	−25.68	< 0.001	809,379	2,021,487
**Length of hospital stay**	−0.4926	0.0226	−21.76	< 0.001	809,379	2,021,487

The average effects are reported in the coefficient column

*Abbreviations*: *CKD* Chronic kidney disease

The effects of medical migration on different groups based on patients’ insurance type were further estimated. We conducted PSM estimations for each group; the graph of common support and the test of covariate balance were reported in Appendix Figs. S2-S5 and Appendix Tables S5-S8. Estimation results showed that, for patients with UBMI, NRCMC, commercial insurance, and self-payment, medical migration was associated with 22.17, 25.00, 29.28, and 35.22% higher medical expenditure, respectively, than non-migrant CKD patients (Table 4), which amounted to 2,039, 2,275, 2,723, and 3,310 yuan in level terms (Appendix Table S3). In addition, migrant patients with all four types of health insurance also had lower in-hospital mortality rates, and shorter length of hospital stay than their non-migrant counterparts. Comparing between different insurance types, the effect of medical migration on reductions of mortality rates are similar across insurance types, amounting to 0.23 percentage point (pp), 0.25 pp., 0.23 pp., and 0.26 pp. for UBMI, NRCMC, commercial insurance, and self-payment, respectively. However, considering that migrant patients with self-payment incurred a statistically significantly larger increase in medical expenditure (35.22%) than their counterparts with public health insurance of UBMI and NRCMC (22.17 and 25.00%), migrant patients with self-payment faced a lower cost-effectiveness in medical migration than their counterparts with public health insurance. Additionally, patients with self-payment and commercial insurance had smaller reductions in length of hospital stay (− 0.0979 and − 0.2307 days) after medical migration than patients with public health insurance of UBMI and NRCMC (− 0.5967 days and − 0.6270 days).

**Table 4:** Estimated effects of medical migration on expenditure and health outcomes of patients with CKD by insurance type

Variable	Coefficient	Standard Error	T-statistics	*P*-value	Obs (migrant)	Obs (non-migrant)
**Pane A: UBMI**						
**Expenditure (log)**	0.2217	0.0027	82.25	< 0.001	178,874	785,942
**Mortality rate**	−0.0023	0.0002	−12.01	< 0.001	178,874	785,942
**Length of hospital stay**	−0.5967	0.0352	−16.95	< 0.001	178,874	785,942
**Panel B: NRCMC**						
**Expenditure (log)**	0.2500	0.0028	89.66	< 0.001	228,033	540,514
**Mortality rate**	−0.0025	0.0002	−12.95	< 0.001	228,033	540,514
**Length of hospital stay**	−0.6270	0.0392	−15.99	< 0.001	228,033	540,514
**Panel C: Commercial Health Insurance**						
**Expenditure (log)**	0.2928	0.0035	83.65	< 0.001	185,683	378,309
**Mortality rate**	−0.0023	0.0002	−11.47	< 0.001	185,683	378,309
**Length of hospital stay**	−0.2307	0.0409	- 5.64	< 0.001	185,683	378,309
**Panel D: Self-payment**						
**Expenditure (log)**	0.3522	0.0038	92.44	< 0.001	217,479	298,083
**Mortality rate**	−0.0026	0.0002	−13.27	< 0.001	217,479	298,083
**Length of hospital stay**	−0.0979	0.0484	−2.02	0.043	217,479	298,083

Panel A reports the results for the sample of patients with UBMI; Panel B for patients with NRCMC; Panel C for patients with other health insurance, such as commercial insurance; Panel D for self-paid patients

*Abbreviations*: *CKD* Chronic kidney disease, *UBMI* Urban basic medical insurance, *NRCMC* New rural co-operative medical care

We also estimated the effect of medical migration on subsample of patients with different demographic characteristics (Appendix Table S9). The effects of medical migration on log medical expenditure were similar between male and female patients, and similar across different age groups. However, the effect on mortality rate was more pronounced among older patients than younger ones. The reductions of mortality rate were 0.13 pp., 0.25 pp., 0.43 pp., and 0.56 pp., among CKD patients aged below 40, 40–60, 60–80, and above 80 years, respectively. In addition, the estimated effects of medical migration on medical expenditure and mortality varied substantially across different origin provinces (Appendix Table S10), reflecting large regional variation in migrant patients’ health conditions, income, and health insurance policies from different regions.

The cost-effectiveness of medical migration was evaluated based on the estimated benefits (reduction in mortality rates) and costs (increase in medical expenditure). Based on the average medical expenditure of 9,129 yuan over the entire baseline sample (as in Table 1), the 26.35% increase in medical expenditure was converted to a monetary increase of 2,405 yuan. The associated average cost per life saved of medical migration was 1,002,288 yuan. Moreover, the cost per life saved for migrant patients with public health insurance (UBMI and NRCMC) was much lower than migrant patients with self-payment and commercial insurance. The associated average cost per life saved of migrant patients with UBMI and NRCMC were similar and were not statistically different from each other, at 879,956 yuan and 912,489 yuan, respectively. In contrast, the average cost per life saved of medical migrants with self-payment was 1,236,628 yuan, or over 35% higher than those with UBMI or NRCMC. The average cost per life saved of medical migrants with commercial insurance was 1,162,161 yuan.

The above measures of cost per life saved for medical migration only accounted for the medical cost, and excluded the additional cost from travelling to and lodging in a non-residential province. We conducted a back-of-the-envelope calculation to account for these additional costs. In our sample, migrant patients on average travelled an additional 312 km (measured as distance between city centroids) than non-migrant patients to seek inpatient care. We assumed a transportation cost of 0.5 yuan per kilometer (a conservative estimate based on the mileage price of the bullet train, higher price if using private transport). Accordingly, migrant patients were estimated to pay an additional 156 yuan on transportation. The average length of stay was 8 days for migrant patients, and compared to non-migrants, the length of stay was reduced by 0.5 days. Therefore, we estimated a net lodging cost of 750 yuan for migrant patients by assuming a daily lodging expense of 100 yuan.^1^ Adding the additional cost of 906 yuan in transportation and lodging to the additional medical expenditure of 2,355 yuan, the combined additional cost per life saved from medical migration amounted to 1.4 million yuan. We further tested the sensitivity of this estimate to different assumptions of transportation cost and lodging cost (Appendix Table S11). Under the transportation cost of 0.25–1.5 yuan per KM and the lodging cost of 50–200 yuan per day, the estimated total cost per life saved would range from 1.22–1.69 million yuan, or within 20% of our baseline estimate of 1.4 million yuan.

The additional cost per life saved represents an incremental cost-effectiveness ratio (ICER), which is a summary measure commonly adopted in cost-effectiveness assessment. In our setting, ICER was calculated by dividing the incremental cost (measured as the change in medical expenditure) by the incremental effect in the measure of health benefits (measured as the reduction in mortality) to provide a ratio of extra cost per unit of health benefits from medical migration. We found that the ICER for medical migration was 1 million yuan per life saved if measuring cost as the medical expenditure, and 1.4 million yuan per life saved if including extra expenditure on transportation and lodging.

## Discussion

We provide one of the first study to delineate the economic burden and cost-effectiveness of medical migration for CKD patients in China, using a large national database of inpatient admission records. Our study suggested that the pattern of medical migration was prominent among CKD patients, and medical migration led to substantially higher financial burden from medical expenditure and various non-medical cost. Therefore, it is necessary for health care policy makers to design a more balanced regional allocation of medical resources, and to lower the financial barriers to trans-provincial medical care seeking.

According to the Report on the Work of the Government 2019, China will thrive to allow patients to use their insurance cards for medical treatment in any designated hospitals across China [26]. The Chinese government hopes to improve the capacity of community medical institutions while making reimbursement of trans-provincial medical expenditure more convenient for migrant patients. Currently, direct reimbursement settlement can be done in more than 41,000 medical institutions in China [27]. Our study showed the percentage of trans-provincial hospitalizations among patients with CKD was rising steadily over years. This situation is essentially associated with China’s rapid economic growth in recent decades, population aging, and unbalanced development of health care system across regions. But it deviates from the intention of promoting the tiered health-care delivery system. Compared with other major non-communicable diseases, large geographic disparities in clinical capacity and quality of care exist in nephrology [10, 28]. Thus, optimizing the allocation of resources and enhancing the capacity and accessibility of kidney care is an emerging policy priority. The establishment of a regional network for more accessible and inclusive management for CKD patients and the design of insurance reimbursement policies that are not restricted by administrative barriers may be effective solutions.

To evaluate the cost-effectiveness of medical migration in China, we compared the estimated cost per life saved to a societal value on the mortality risk reduction. A standard approach to placing a monetary value on the mortality risk reduction is to estimate the societal willingness to pay (WTP) to save a “statistical life”. The value of a statistical life (VSL) measures the additional monetary value that individuals are willing to pay to reduce the mortality risk and is frequently used to monetize health benefits of health care regulations.

Extensive VSL studies have been conducted in the developed countries, but relatively few can be found in developing countries. Most existing studies adopted the contingent evaluation (CV) method, which is the most widely used method in the recent literature.^2^ It is a stated preference method that elicits an individual’s WTP for a specified mortality risk reduction, commonly elicited through individual interviews or hypothetical choice experiments, and computes the VSL by dividing the change in WTP by the associated reduction in mortality risks. This CV method is a natural match to our research design.

We reviewed several contemporaneous VSL studies in China to obtain an up-to-date estimate of the value of VSL [29–35]. For the ease of comparison, we adjusted all the estimates of VSL to constant 2016 US dollar using inflation adjustment and exchange rate adjustment.^3^ In general, VSL estimates from more recent studies were substantially larger than those from earlier studies. Hammitt et al. attributed this substantial increase in estimated VSL to the rapid increase of real income in China [33]. They found the estimated VSL increased from $22,000 (144,320 yuan) in 2005 to $550,000 (3.61 million yuan) in 2016. Most recently, Cao et al. conducted interviews in six representative Chinese cities in 2019 and found a VSL range of $474,728–$944,046 and a mean VSL of $708,711, or equivalently, 4.90 million yuan adjusted to 2016 constant price [34]. It is noteworthy that existing estimates of VSL vary widely across studies due to differences in study population’s demographics and socioeconomic status. However, despite the unavailability of a national estimate of VSL, the two most recent studies have presented rather close estimates of VSL—3.61 million yuan and 4.90 million yuan, respectively, which serve as rough bounds for a national estimate of VSL in recent years.

Our estimated average cost per life saved of medical migration is lower than the median of contemporaneous estimates of VSL in existing studies. Taken the two most recent estimates of VSL as the benchmark, our estimated cost per life saved of medical migration (1 million yuan) accounts for 20–27% of VSL; if the cost includes the additional expenditure from trans-provincial transportation and lodging, the cost per life saved (1.4 million yuan) accounts for 29–39% of VSL. This implies that CKD patients’ average willingness to pay for a reduction in mortality outweighs the additional cost associated with trans-provincial medical migration.

Although our results imply that medical migration could be a cost-effective means for seeking better kidney care in terms of VSL, medical migration nevertheless led to a substantial increase in financial burden for CKD patients and their families. This additional cost can be attributed to the administrative barriers in reimbursement policies on trans-provincial medical care utilization. The elimination of administrative barriers between different regional health care systems could help reduce the economic burden of CKD patients. Our findings shed light on the design of future health insurance policies from a macro point-of-view.

We proceed to assess the cost-effectiveness of medical migration for patients with different health insurance schemes. Since the reimbursement rate varies under different health insurance scheme, the potential *out-of-pocket* cost per life saved may be different for migrant patients with different insurance schemes. We found that the cost per life saved for migrant patients with public health insurance of UBMI and NRCMC (879,956 yuan and 912,489 yuan) was much lower than migrant patients with self-payment and commercial insurance (1,236,628 yuan and 1,162,161 yuan). Considering that both UBMI and NRCMC have some cost-sharing for patients hospitalized outside of their registered region, the *out-of-pocket* costs per life saved for UBMI- and NRCMC-insured medical migrants are even lower than that for the self-paid. This suggests that public health insurance reduce CKD patients’ cost of medical care seeking across provincial borders.

Our study has the advantages of using a large national database, strict quality control measures to ensure data validity and reliability. However, there are certain limitations which deserve mention. Firstly, although our data are restricted to hospitalized patients, hospitalization inherently reflects indication for admission and referral as well as disease burden. Only tertiary hospitals were included in our analyses. Secondly, the diagnosis of CKD in hospitalized patients was based on the ICD-10 coding with low sensitivity and severe cases of CKD were likely to be diagnosed, potentially leading to a selection bias. Thirdly, although we have included some estimates of transportation and accommodation cost in the cost-effectiveness analysis, the current assessment might not fully incorporate the non-medical cost of medical migration. We thus interpreted our estimate as a lower bound for the overall financial burden. Finally, data of kidney function or proteinuria, information on medications used, utilization of care in community health facilities, and out-of-hospital death were not available in our dataset.

## Conclusions

Our study evaluates the cost-effectiveness of medical migration among CKD patients in China, and finds substantial financial burden for migrant patients. It is imperative to reduce the regional disparity in allocation of medical resources and service capacity. Our study provides real-world supporting evidence to policies that prioritize resource allocation toward expanding kidney health care capacity and establishing regional network of kidney care and insurance plans. More research is needed to uncover factors that drive cross-regional health-seeking behaviors of CKD patients and patients with other chronic diseases.

## Supplementary Information

**Additional file 1.**

## Data Availability

The data that support the findings of this study are available from the Bureau of Medical Administration and Medical Service Supervision, National Health Commission of China but restrictions apply to the availability of these data, which were used under license for the current study, and so are not publicly available. Data are however available from the authors upon reasonable request and with permission of the Bureau of Medical Administration and Medical Service Supervision, National Health Commission of China.

## Abbreviations

CKD: Chronic kidney disease
CVD: Cardiovascular disease
ESKD: End-stage kidney disease
HQMS: Hospital Quality Monitoring System
ICD-10: International Classification of Diseases, Tenth Revision
IQR: Inter-quartile range
KRT: Kidney replacement therapy
NHC: National Health Commission
NRCMC: New rural co-operative medical care
PSM: Propensity score method
SD: Standard deviation
UBMI: Urban basic medical insurance
VSL: Value of statistical life

## Footnotes

1 This is a conservative estimate because most migrant patients travelled to large cities, where cost of living was high and the daily hotel prices were much higher than 100 yuan. This calculation also assumes no accompanying family member who cared for the migrant patient, as we have no such information. Had one or more family members travelled with the migrant patient to the distant province, the additional cost from transportation and lodging would be even higher.

2 Alternatively, a number of earlier studies in developed countries adopted the method of wage differentials to estimate VSL, which measures compensating wage differences for on-the-job risk exposures in competitive labor markets. Specifically, this method regresses the average wage of different occupations on the occupational fatality risk. This method is yet to be widely adopted to measure VSL in developing countries due to its requirement of a competitive labor market, reliable estimates of occupational mortality risks and average wage across occupations.

3 Although none of existing VSL studies in China were designed specifically for CKD, most of them have designed their interviews or experiments to elicit WTP to reduce disease-related morbidity and mortality risks, such as the mortality risk from all-cause cancer. These estimates of VSL serve as a benchmark for VSL in CKD-related scenarios.
