# Supplementary material for: Cost-effectiveness of medical migration for chronic kidney disease: a national cross-sectional study in China
Source: BMC Health Serv Res. 2022 Jul 13;22:912. doi: 10.1186/s12913-022-08266-x (PMC9281168; doi:10.1186/s12913-022-08266-x)
Supplement: Supplementary file 1 — Additional file 1. [file 12913_2022_8266_MOESM1_ESM.docx]

# Appendix A: Brief review of recent VSL literature in China

Wang and Mullahy (2006) carried out a CV study based on interviews conducted in 1998 to estimate WTP for reducing the risk of fatality due to air pollution. They estimated the median value of a Chinese statistical life to be $150,619. Hammitt and Zhou (2006) conducted a CV study based on air-pollution-related health risks and estimated a value of VSL ranging from $150,000 to $200,000 in Beijing. Wang and He (2014) estimated the VSL based on surveys conducted in 2000 to elicit individuals’ WTP to reduce all cause cancer morbidity and mortality risks and find an estimate of VSL ranging from $246,859 to $417,761. More recently, Ohdoko, Komatzu and Kaneko (2013) conducted choice experiments in 2008 about air pollution related cancer risks and estimated a VSL range of $1,255,923 to $1,672,901. Based on similar air pollution related disease scenarios, Huang, Andersson and Zhang (2017) conducted CV surveys in 2010 and estimated VSL to be $544,386 to $1,082,570.

In general, VSL estimates from more recent studies were substantially larger than those from earlier studies. Hammitt, Geng, Guo, and Nielsen (2019) attributed this substantial increase in estimated VSL to the rapid increase of real income in China in recent decade. They found the estimated VSL increased from $22,000 (144,320 yuan) in 2005 to $550,000 (3.61 million yuan) in 2016. Most recently, Cao, Song, et al. (2021) conducted interviews in six representative Chinese cities in 2019 and found a VSL range of $474,728-$944,046 and a mean VSL of $708,711, or equivalently, 4.90 million yuan adjusted to 2016 constant price. For our cost-effectiveness analysis, we adopt these two most recent estimates of VSL—3.61 million yuan from Hammitt, Geng, Guo, and Nielsen (2019) and 4.90 million yuan from—as our baseline estimates of VSL.

To adopt existing estimates of VSL in our analysis faces a limitation: The existing VSL estimates vary widely across studies. Such variation can be attributed to the sample differences in demographic and socioeconomic status across study regions as well as the dramatic increase in average income over time in China. However, despite the unavailability of a national estimate of VSL, the two recent studies have presented rather close estimates of VSL, which may serve as a rough bound for a national average of VSL in recent years.

Although the estimates of VSL in China are largely dependent on the estimation methods and sampled population, the estimated average cost per life saved of medical migration is generally lower than the range of contemporaneous estimates of VSL in recent studies. In particular, the average medical cost per life saved of medical migration (1,002,288 yuan) accounts for 20-27 percent of societal value of a statistical life; if the cost includes the additional expenditure from trans-provincial transportation and lodging, the cost per life saved accounts for 29-39 percent of VSL. This means that CKD patients’ average willingness to pay for the reduction in mortality risks outweighs the additional cost associated with trans-provincial medical migration. This explains the rising trend of CKD medical migration in recent decade.

Although our results imply that medical migration is cost-effective compared to the large value of VSL, medical migration is nevertheless associated with a substantial increase in medical expenditure. This additional cost can be partially attributed to the administrative barriers in reimbursement policies on trans-provincial medical care utilization. The elimination of administrative barriers between different health systems could help reduce the economic burden of CKD patients. Our findings provide insights for development of insurance policies from a macro point-of-view.

Since the reimbursement rate varies for migrant patients under different health insurance scheme, the potential *out-of-pocket* cost per life saved may be different across insurance schemes. We found that the cost per life saved for migrant patients with public health insurance (879,956 yuan and 912,489 yuan for UBMI and NRCMC, respectively) was much lower than migrant patients with self-payment (1,236,628 yuan). Considering that both UBMI and NRCMC have cost-sharing for patients hospitalized outside of their registered region, the *out-of-pocket* costs per life saved for UBMI- and NRCMC-insured medical migrants are even lower than that for the self-paid. This suggests that public health insurance reduce CKD patients’ cost of medical care seeking across provincial borders.

# Appendix B: Tables

**Table S1. ICD-10 Coding of CKD Etiologies**

| Etiology of CKD | All editions | China edition | Beijing edition | Clinic edition |
| --- | --- | --- | --- | --- |
| 1. Diabetes mellitus |  |  |  |  |
| Type 1 diabetes mellitus with renal complications | E10.2+ N08.3 |  |  |  |
| Type 2 diabetes mellitus with renal complications | E11.2+ N08.3 |  |  |  |
| Unspecified diabetes mellitus with renal complications | E14.2 |  |  |  |
| Malnutrition-related diabetes mellitus with renal complications |  | E12.200+N08.3 |  | E12.200 |
| Other specified diabetes mellitus with renal complications |  | E13.2 |  | E13.200 |
| 2. Hypertensive diseases |  |  |  |  |
| Hypertensive renal disease with renal failure | I12 |  |  |  |
| Hypertensive heart and renal disease with (congestive) heart failure | I13 |  |  |  |
| Pregnancy with hypertensive heart and renal disease | O10.301 |  |  |  |
| Pregnancy with essential hypertension and proteinuria | O11.x01 |  |  |  |
| Pre-existing hypertensive renal disease during pregnancy, childbirth and puerperium |  | O10.200 |  | O10.200 |
| Pregnancy with hypertensive renal disease |  | O10.201 |  | O10.201 |
| Pre-existing hypertensive heart and renal disease during pregnancy, childbirth and puerperium |  | O10.300 |  | O10.300 |
| Pre-existing hypertension with proteinuria |  | O11.x00 |  | O11.x00 |
| 3. Glomerular diseases |  |  |  |  |
| Recurrent and persistent hematuria | N02 |  |  |  |
| Chronic nephritic syndrome | N03 |  |  |  |
| Nephrotic syndrome | N04 |  |  |  |
| Unspecified nephritic syndrome | N05 |  |  |  |
| Isolated proteinuria with specified morphological lesion | N06 |  |  |  |
| Persistent proteinuria, unspecified | N39.1 |  |  |  |
| 4. Renal tubulointerstitial diseases |  |  |  |  |
| Chronic tubulointerstitial nephritis | N11 |  |  |  |
| Tubulointerstitial nephritis, not specified as acute or chronic | N12 |  |  |  |
| Drug- and heavy-metal-induced tubulointerstitial and tubular conditions | N14 |  |  |  |
| Renal tubulointerstitial disorders in diseases classified elsewhere | N16 |  |  |  |
| Other specified disorders of carbohydrate metabolism | E74.8 |  |  |  |
| Disorders of amino-acid transport | E72.0 |  |  |  |
| Nephrogenic diabetes insipidus |  | N25.1 |  | N25.1 |
| Renal tubule acidosis | N25.8 |  |  |  |
| Balkan nephropathy |  | N15.000 | N15.001 | N15.000 |
| Renal tubulointerstitial disease, specified |  | N15.800 |  | N15.800 |
| Renal granuloma |  | N15.801 |  | N15.801 |
| Renal tubulointerstitial disease |  | N15.900 |  | N15.900 |
| Impaired renal tubular function-related disease |  | N25.9 |  | N25.9 |
| Liddle syndrome |  | I15.101 |  | I15.101 |
| Urate nephropathy |  | M10.001+N16.8 | N28.905 | M10.001+N16.8 |
| Systemic lupus erythematosus + renal tubulointerstitial diseases |  | M32.102+N16.4 | M32.113+N16.4 | M32.102+N16.4 |
| Sicca syndrome + renal tubulointerstitial diseases |  | M35.006+N16.4 | M35.005+N16.4 | M35.006+N16.4 |
| 5. Obstructive nephropathy |  |  |  |  |
| Hydronephrosis with ureteropelvic junction obstruction | N13.0 |  |  |  |
| Hydronephrosis with ureteral stricture, not elsewhere classified | N13.1 |  |  |  |
| Hydronephrosis with renal and ureteral calculous obstruction |  | N13.2 | N13.2 | N13.200 |
| Other obstructive nephropathy |  | N13.8 | N13.8 | N13.801 |
| 6. Other related diagnosis |  |  |  |  |
| Hereditary nephropathy, not elsewhere classified |  | N07 | N07.901 | N07 |
| Glomerular disorders in diseases classified elsewhere | N08, exclude N08.5 |  |  |  |
| Renal agenesis and other reduction defects of kidney | Q60 |  |  |  |
| Polycystic kidney, autosomal recessive | Q61.1 |  |  |  |
| Polycystic kidney, autosomal dominant | Q61.2 |  |  |  |
| Polycystic kidney, unspecified | Q61.3 |  |  |  |
| Medullary cystic kidney, Sponge kidney NOS | Q61.5 |  |  |  |
| Lobulated, fused and horseshoe kidney | Q63.1 |  |  |  |
| Congenital malformation of kidney, unspecified | Q63.9 |  |  |  |
| Gout due to impairment of renal function |  | M10.300 | M10.393 | M10.300 |
| Unspecified contracted kidney | N26 |  |  |  |
| Ischaemia and infarction of kidney | N28.0 |  |  |  |
| Other specified disorders of kidney and ureter | N28.8 |  |  |  |
| Disorders of kidney and ureter, unspecified | N28.9 |  |  |  |
| Congenital renal failure |  | P96.0 | P96.0 | P96.000 |
| Extrarenal uraemia | R39.2 |  |  |  |
| Aortic arch syndrome + renovascular hypertension |  | M31.4 + I15.0 | I77.604 + I15.0 | I77.600x004 + I15.0 |
| Goodpasture syndrome | M31.001 |  |  |  |
| Renal osteodystrophy | N25.0 |  |  |  |
| Failure and rejection of renal transplantation | T86.1 |  |  |  |
| Hemolytic uraemic syndrome | D59.3 |  |  |  |
| Dialysis | Z49 |  |  |  |
| Renal allergic purpura | D69.005+N08.2 |  |  |  |
| Lupus nephritis |  | M32.101+N08.5 | M32.105+N08.5 | M32.101+N08.5 |
| Goodpasture syndrome-related glomerulonephritis |  | M31.003+N08.5 |  | M31.003+N08.5 |
| Antiglomerular basement membrane antibody-related disease |  | M31.002+N08.5 | M31.005+N08.5 | M31.002+N08.5 |
| Microscopic polyangitis |  | M31.700 | M31.701 | M31.700 |
| ANCA-related nephritis |  | M31.701+N08.5 | M31.802 | M31.701+N08.5 |
| Thrombotic thrombocytopenic purpura-related glomerulonephritis |  | M31.102+N08.5 |  | M31.102+N08.5 |
| Wegener’s granulomatosis-related glomerulonephritis |  | M31.303+N08.5 |  | M31.303+N08.5 |
| Pregnancy with nephrotic syndrome |  | O26.801 | O26.811 | O26.801 |
| Pregnancy with glomerulonephritis |  | O26.804 | O26.812 | O26.804 |
| Pregnancy with renal failure |  | O26.802 | O26.813 | O26.802 |
| HBV-related nephritis |  | B18.103+N08.0 | B18.102 | B18.103+N08.0 |
| HCV-related nephritis |  | B18.205+N08.0 | B18.208 | B18.205+N08.0 |
| Cryoglobulinaemia-related glomerulonephritis |  | D89.101+N08.2 |  | D89.101+N08.2 |
| Hereditary amyloidosis nephropathy |  | E85.002 | E85.003 | E85.002 |
| Amyloidosis-related nephropathy |  | E85.411+N29.8 | E85.410+N08.4 | E85.411+N29.8 |
| Psoriatic nephritis |  | L40.803+ | L40.802+N05.9 | L40.800x002+N05.9 |
| Kidney injury-related gout |  | M10.300 | M10.393 | M10.300 |
| Syphilitic nephritis |  |  | A52.712+N08.0 | A52.700x012+N08.0 |
| Lupus kidney injury |  |  | M32.112+N08.5 |  |
| Lupus nephritis |  | M32.101+N08.5 | M32.105+N08.5 | M32.101+N08.5 |
| Lupus tubulointerstitial kidney |  | M32.102+N16.4 | M32.113+N16.4 | M32.102+N16.4 |
| Gouty nephropathy |  |  | M10.391 | M10.300x091 |
| Gouty nephrolithiasis |  | M10.005+N22.8 | M10.392 | M10.005+N22.8 |

Abbreviations: ANCA, antineutrophil cytoplasmic antibodies; CKD, chronic kidney disease; HBV, hepatitis B virus; HCV, hepatitis C virus; ICD, International Classification of Diseases, Tenth Revision.

**Table S2. ICD-10 Coding of CVD, diabetes and hypertension**

| Comorbidity | All editions | China edition | Beijing edition | Clinic edition |
| --- | --- | --- | --- | --- |
| CVD |  |  |  |  |
| 1. Cerebral stroke |  |  |  |  |
| Subarachnoid hemorrhage | I60 |  |  |  |
| Intracerebral hemorrhage | I61 |  |  |  |
| Acute ischemic cerebral stroke | I63 |  |  |  |
|  | I64 |  |  |  |
|  | H34.1 |  |  |  |
| Transient ischemic attack | G45 |  |  |  |
| 2. Coronary heart disease |  |  |  |  |
| Angina pectoris | I20 |  |  |  |
| Acute myocardial infarction | I21 |  |  |  |
| Subacute myocardial infarction | I22 |  |  |  |
| Complications after myocardial infarction | I23 |  |  |  |
| Other acute ischemic heart disease | I24 |  |  |  |
| Chronic ischemic heart disease | I25 |  |  |  |
| 3. Heart failure |  |  |  |  |
| Whole-hearted failure |  |  | I50.003 | I50.002 |
| Right heart failure |  | I50.001 | I50.004 | I50.001 |
| Right ventricular failure |  | I50.005 |  | I50.000x005 |
| Acute right heart failure |  |  |  | I50.000x006 |
| Left heart failure |  | I50.100 | I50.106 | I50.100x006 |
| Left ventricular failure |  |  |  | I50.100 |
| Left atrial failure |  |  |  | I50.102 |
| Chronic left heart insufficiency |  |  | I50.103 | I50.105 |
| Left heart failure with acute pulmonary edema |  |  | I50.107 | I50.103 |
| Congestive heart failure |  | I50.000 | I50.001 | I50.000 |
| Acute heart failure |  |  | I50.904 | I50.907 |
| Chronic heart failure |  |  | I50.905 | I50.908 |
| Other heart failure |  | I50.900 | I50.911 | I50.900 |
| Postoperative heart failure and pulmonary edema |  |  | I97.104 | I97.100x004 |
| Heart failure of newborns |  | P29.000 | P29.001 | P29.000 |
| Hypertensive heart failure |  |  |  | I11.001 |
| Hypertensive heart disease with (congestive) heart failure |  | I11.000 |  | I11.000 |
| Hypertensive heart disease without (congestive) heart failure |  |  |  | I11.900 |
| Hypertensive heart disease and kidney disease with congestive heart failure |  | I13.000 |  | I13.000 |
| Hypertensive heart disease and kidney disease with congestive heart failure and renal failure |  | I13.200 |  | I13.200 |
| Intractable heart failure |  |  |  | I50.900x017 |
| Heart failure after cardiac surgery |  | I97.102 | I97.106 | I97.102 |
| Postoperative heart failure |  | I97.803 |  | I97.803 |
| Chronic left heart insufficiency |  |  | I50.103 | I50.105 |
| Cardiac insufficiency |  | I50.901 | I50.902 | I50.900x002 |
| Cardiac insufficiency of newborns |  | P29.001 |  |  |
| Acute exacerbation of chronic cardiac insufficiency |  |  |  | I50.900x018 |
| Acute left heart failure |  |  | I50.102 | I50.101 |
| Acute pulmonary edema |  |  | J81xx02 | J81.x00x002 |
| Pregnancy with heart failure |  | O99.417 | O99.408 | O99.400x008 |
| Pregnancy with cardiac insufficiency |  | O99.429 | O99.429 | O99.414 |
| Childbirth with heart failure |  | O75.403 |  | O75.403 |
| Pregnancy with left heart failure |  |  | O99.423 | O99.424 |
| Puerperal cardiac insufficiency |  | O99.402 | O99.434 | O99.402 |
| Acute pulmonary edema after postpartum |  | O99.507 | O99.508 | O99.508 |
| Heart failure due to anesthesia during pregnancy |  |  | O29.102 | O99.500x008 |
| Heart failure due to anesthesia during childbirth |  |  | O74.202 | O74.200x002 |
| Heart failure after obstetric surgery or operation |  |  | O75.402 |  |
| Heart failure due to anesthesia during puerperium |  |  | O89.102 | O89.100x002 |
| Low cardiac output syndrome |  | I50.901 | I50.901 |  |
| Cardiac function, class I |  | I50.902 | I50.902 |  |
| Cardiac function, class II |  | I50.903 | I50.907 | I50.903 |
| Cardiac function, class III |  | I50.904 | I50.908 | I50.904 |
| Cardiac function, class IV |  | I50.905 | I50.910 | I50.905 |
| Cardiac function, class II (NYHA) |  |  |  | I50.900x007 |
| Cardiac function, class III (NYHA) |  |  |  | I50.900x008 |
| Cardiac function, class II~III (NYHA) |  |  |  | I50.900x009 |
| Cardiac function, class IV(NYHA) |  |  |  | I50.900x010 |
| Circulatory failure |  | R57.901 | I50.913 | R57.901 |
| Pulmonary edema |  | J81.x00 | J81xx03 | J81.x00 |
| Cardiogenic shock |  | R57.000 | R57.001 | R57.000 |
| Respiratory and circulatory failure |  |  | J96.102 | J96.900 |
| Cardiogenic asthma |  |  | I50.104 | I50.104 |
| 4. Atrial fibrillation |  |  |  |  |
| Atrial fibrillation |  | I48.x01 | I48xx04 | I48.x01 |
| Idiopathic atrial fibrillation |  | I48.x02 | I48xx02 | I48.x05 |
| Persistent atrial fibrillation |  |  | I48xx07 | I48.x00x007 |
| Chronic atrial fibrillation |  |  | I48xx08 | I48.x00x008 |
| Pregnancy with atrial fibrillation |  | O99.427 | O99.427 | O99.400x027 |
| Atrial fibrillation with flutter |  | I48.x00 | I48xx01 | I48.x00 |
| Primary atrial fibrillation |  |  |  | I48.x00x009 |
| Long-term persistent atrial fibrillation |  |  |  | I48.x00x011 |
| Acute atrial fibrillation |  |  |  | I48.x00x012 |
| Permanent atrial fibrillation |  |  |  | I48.x00x013 |
| Long-range persistent atrial fibrillation |  |  |  | I48.x00x014 |
| New diagnosis of atrial fibrillation |  |  |  | I48.x00x015 |
| Paroxysmal atrial fibrillation |  | I48.x02 | I48xx06 | I48.x02 |
| Diabetes |  |  |  |  |
| Type 1 diabetes mellitus | E10 |  |  |  |
| Type 2 diabetes mellitus | E11 |  |  |  |
| Malnutrition-related diabetes mellitus | E12 |  |  |  |
| Other specified diabetes mellitus | E13 |  |  |  |
| Unspecified diabetes mellitus | E14 |  |  |  |
| Hypertension |  |  |  |  |
| Essential (primary) hypertension | I10 |  |  |  |
| Hypertensive heart disease | I11 |  |  |  |
| Hypertensive renal disease | I12 |  |  |  |
| Hypertensive heart and renal disease | I13 |  |  |  |
| Secondary hypertension | I15 |  |  |  |

Abbreviations: CVD, cardiovascular disease; ICD, International Classification of Diseases, Tenth Revision; NYHA, New York Heart Association.

**Table S3. Estimated effects of medical migration on medical expenditure of CKD patients**

|  | (1) | (2) | (3) | (4) | (5) | (6) |
| --- | --- | --- | --- | --- | --- | --- |
|  | Coefficient | Standard Error | T-statistics | *p*-value | Obs (migrant) | Obs  (non-migrant) |
| Panel A: Full sample | | | | | | |
| Expenditure (RMB) | 2578 | 16.55 | 155.74 | 0.000 | 809,379 | 2,021,487 |
| Panel B: UBMI sample | | | | | | |
| Expenditure (RMB) | 2039 | 23.94 | 85.19 | 0.000 | 178,874 | 785,942 |
| Panel C: NRCMC sample | | | | | | |
| Expenditure (RMB) | 2275 | 27.46 | 82.83 | 0.000 | 228,033 | 540,514 |
| Panel D: Commercial insurance sample | | | | | | |
| Expenditure (RMB) | 2723 | 30.69 | 88.72 | 0.000 | 185,683 | 378,309 |
| Panel E: Self-payment sample | | | | | | |
| Expenditure (RMB) | 3310 | 36.69 | 90.23 | 0.000 | 217,479 | 298,083 |

Abbreviations: CKD, chronic kidney disease; CVD, cardiovascular disease; NRCMC, new rural co-operative medical care; UBMI, urban basic medical insurance.

**Table S4. Estimated effects of medical migration on expenditure and health outcomes of patients with CKD**

|  | (1) | (2) | (3) | (4) | (5) | (6) |
| --- | --- | --- | --- | --- | --- | --- |
|  | Coefficient | Standard Error | T-statistics | *p*-value | Obs (migrant) | Obs  (non-migrant) |
| Panel A: Baseline: nearest neighbor matching with ties and a caliper of 0.1 | | | | | | |
| Expenditure (log) | 0.2635 | 0.0016 | 166.74 | 0.000 | 809,379 | 2,021,487 |
| Mortality rate | -0.0024 | 0.0001 | -25.68 | 0.000 | 809,379 | 2,021,487 |
| Length of hospital stay | -0.4926 | 0.0226 | -21.76 | 0.000 | 809,379 | 2,021,487 |
| Panel B: Alternative: nearest neighbor with one-to-one matching and a caliper of 0.1 | | | | | | |
| Expenditure (log) | 0.2571 | 0.0033 | 77.91 | 0.000 | 809,379 | 809,379 |
| Mortality rate | -0.0024 | 0.0003 | -8.00 | 0.000 | 809,379 | 809,379 |
| Length of hospital stay | -0.5010 | 0.0390 | -12.85 | 0.000 | 809,379 | 809,379 |
| Panel C: Alternative: nearest neighbor matching with ties and a caliper of 0.01 | | | | | | |
| Expenditure (log) | 0.2610 | 0.0025 | 104.42 | 0.000 | 789,610 | 1,719,601 |
| Mortality rate | -0.0023 | 0.0002 | -11.50 | 0.000 | 789,610 | 1,719,601 |
| Length of hospital stay | -0.4843 | 0.0306 | -15.83 | 0.000 | 789,610 | 1,719,601 |
| Panel D: Alternative: nearest neighbor matching one-to-one matching and a caliper of 0.01 | | | | | | |
| Expenditure (log) | 0.2598 | 0.0035 | 74.23 | 0.000 | 789,235 | 789,235 |
| Mortality rate | -0.0023 | 0.0003 | -7.67 | 0.000 | 789,235 | 789,235 |
| Length of hospital stay | -0.4681 | 0.0421 | -11.12 | 0.000 | 789,235 | 789,235 |

Abbreviations: CKD, chronic kidney disease.

Note: The average effects are reported in the coefficient column. Panel A reports the baseline results based on nearest neighbor matching with tied pairs and a caliber of 0.1; Panel B reports the results based on nearest neighbor matching with 1-to-1 matching pairs and a caliber of 0.1; Panel C reports the results based on nearest neighbor matching with tied pairs and a caliber of 0.01.

**Table S5. Test of the covariate balance of UBMI sample after PSM estimation**

|  | Mean | | T-test | |
| --- | --- | --- | --- | --- |
| Variable | **Migrant** | **Non-migrant** | **T-statistics** | ***P*-value** |
| Male | 0.600 | 0.600 | -0.140 | 0.889 |
| Age≤40 y | 0.293 | 0.293 | -0.190 | 0.846 |
| 40 y<Age≤60 y | 0.480 | 0.479 | 0.090 | 0.931 |
| 60 y<Age≤80 y | 0.221 | 0.221 | -0.020 | 0.984 |
| Hypertension | 0.466 | 0.466 | -0.110 | 0.909 |
| Diabetes | 0.189 | 0.189 | 0.080 | 0.939 |
| CVD | 0.124 | 0.124 | 0.480 | 0.630 |

Abbreviations: PSM, propensity score matching; CVD, cardiovascular disease; UBMI, urban basic medical insurance.

**Table S6. Test of the covariate balance of NRCMC sample after PSM estimation**

|  | Mean | | | T-test | |
| --- | --- | --- | --- | --- | --- |
| Variable | **Migrant** | **Non-migrant** | | **T-statistics** | ***P*-value** |
| Male | 0.534 | | 0.534 | 0.230 | 0.817 |
| Age≤40 y | 0.309 | | 0.310 | -0.270 | 0.790 |
| 40 y<Age≤60 y | 0.463 | | 0.463 | -0.060 | 0.955 |
| 60 y<Age≤80 y | 0.219 | | 0.219 | 0.020 | 0.986 |
| Hypertension | 0.396 | | 0.396 | 0.030 | 0.976 |
| Diabetes | 0.144 | | 0.144 | 0.300 | 0.768 |
| CVD | 0.118 | | 0.117 | 0.530 | 0.594 |

Abbreviations: PSM, propensity score matching; CVD, cardiovascular disease; NRCMS, new rural co-operative medical care.

**Table S7. Test of the covariate balance of self-paid sample of PSM estimation**

|  | Mean | | T-test | |
| --- | --- | --- | --- | --- |
| Variable | **Migrant** | **Non-migrant** | **T-statistics** | ***P*-value** |
| Male | 0.566 | 0.566 | -0.200 | 0.838 |
| Age≤40 y | 0.305 | 0.305 | 0.500 | 0.614 |
| 40 y<Age≤60 y | 0.441 | 0.441 | -0.200 | 0.838 |
| 60 y<Age≤80 y | 0.237 | 0.238 | -0.580 | 0.561 |
| Hypertension | 0.418 | 0.418 | -0.480 | 0.632 |
| Diabetes | 0.173 | 0.172 | 0.100 | 0.920 |
| CVD | 0.119 | 0.117 | 1.580 | 0.113 |

Abbreviations: PSM, propensity score matching; CHD, coronary heart disease; CVD, cardiovascular disease.

**Table S8. Test of the covariate balance of sample with commercial insurance after PSM estimation**

|  | Mean | | T-test | |
| --- | --- | --- | --- | --- |
| Variable | **Migrant** | **Non-migrant** | **T-statistics** | ***P*-value** |
| Male | 0.568 | 0.572 | -0.100 | 0. 920 |
| Age≤40 y | 0.349 | 0.348 | 0.190 | 0. 849 |
| 40 y<Age≤60 y | 0.430 | 0.435 | -0.120 | 0. 904 |
| 60 y<Age≤80 y | 0.227 | 0.228 | -0.496 | 0. 620 |
| Hypertension | 0.398 | 0.402 | -0.180 | 0. 857 |
| Diabetes | 0.145 | 0.151 | 0.323 | 0. 746 |
| CVD | 0.133 | 0.127 | 1.320 | 0. 187 |

Abbreviations: PSM, propensity score matching; CHD, coronary heart disease; CVD, cardiovascular disease.

**Table S9. Sensitivity analyses based on different subpopulations of CKD patients**

|  | Coefficient | Standard Error | T-statistics | *p*-value | Obs (migrant) | Obs  (non-migrant) | |
| --- | --- | --- | --- | --- | --- | --- | --- |
| Panel A: Female | | | | | | | |
| Expenditure (log) | 0.2579 | 0.0023 | 112.13 | 0.000 | 352,889 | 881,368 | |
| Mortality rate | -0.0025 | 0.0002 | -12.54 | 0.000 | 352,889 | 881,368 | |
| Length of hospital stay | -0.5026 | 0.0246 | -20.43 | 0.000 | 352,889 | 881,368 | |
| Panel B: Male | | | | | | |  |
| Expenditure (log) | 0.2702 | 0.0016 | 168.87 | 0.000 | 456,489 | 1,140,118 | |
| Mortality rate | -0.0026 | 0.0001 | -26.23 | 0.000 | 456,489 | 1,140,118 | |
| Length of hospital stay | -0.4626 | 0.0201 | -23.01 | 0.000 | 456,489 | 1,140,118 | |
| Pane C: Age below 40 | | | | | | | |
| Expenditure (log) | 0.2323 | 0.0027 | 82.25 | 0.000 | 239,590 | 598,360 | |
| Mortality rate | -0.0013 | 0.0001 | -12.01 | 0.000 | 239,590 | 598,360 | |
| Length of hospital stay | -0.0934 | 0.0210 | -16.95 | 0.000 | 239,590 | 598,360 | |
| Panel D: Age between 40 and 60 | | | | | | |  |
| Expenditure (log) | 0.2876 | 0.0028 | 89.66 | 0.000 | 310,695 | 825,841 | |
| Mortality rate | -0.0025 | 0.0001 | -12.95 | 0.000 | 310,695 | 540,514 | |
| Length of hospital stay | -0.3170 | 0.0419 | -15.99 | 0.000 | 310,695 | 540,514 | |
| Panel E: Age between 60 and 80 | | | | | | | |
| Expenditure (log) | 0.2509 | 0.0035 | 83.65 | 0.000 | 195,420 | 478,439 | |
| Mortality rate | -0.0043 | 0.0002 | -11.47 | 0.000 | 195,420 | 478,439 | |
| Length of hospital stay | -0.4903 | 0.0512 | - 5.64 | 0.000 | 195,420 | 478,439 | |
| Panel F: Age over 80 | | | | | | |  |
| Expenditure (log) | 0.2923 | 0.0138 | 21.18 | 0.000 | 10,712 | 27,257 | |
| Mortality rate | -0.0056 | 0.0009 | -6.22 | 0.000 | 10,712 | 27,257 | |
| Length of hospital stay | -0.6145 | 0.0932 | -6.59 | 0.000 | 10,712 | 27,257 | |

Abbreviations: CKD, chronic kidney disease.

**Table S10. Sensitivity analyses of the effects of medical migration based on different origin provinces of CKD medical migrants**

| \| **Province Name** \| **Variable** \| **Coefficient** \| **Standard Error** \| **T-stats** \| **p-value** \| **Migrant (obs)** \| **Nonmigrant (obs)** \| \| --- \| --- \| --- \| --- \| --- \| --- \| --- \| --- \| \| **HEBEI** \| Expen. (log) \| 0.330 \| 0.026 \| 12.475 \| 0.000 \| 13,222 \| 72,477 \| \| **HEBEI** \| Mortality \| -0.002 \| 0.002 \| -1.027 \| 0.305 \| 13,222 \| 72,477 \| \| **SHANXI** \| Expen. (log) \| 0.308 \| 0.077 \| 4.018 \| 0.000 \| 13,561 \| 55,023 \| \| **SHANXI** \| Mortality \| -0.002 \| 0.004 \| -0.419 \| 0.675 \| 13,561 \| 55,023 \| \| **INNER MOGOLIA** \| Expen. (log) \| 0.351 \| 0.086 \| 4.058 \| 0.000 \| 7,219 \| 71,661 \| \| **INNER MOGOLIA** \| Mortality \| -0.009 \| 0.007 \| -1.376 \| 0.169 \| 7,219 \| 71,661 \| \| **LIAONING** \| Expen. (log) \| 0.289 \| 0.036 \| 8.096 \| 0.000 \| 11,031 \| 113,141 \| \| **LIAONING** \| Mortality \| -0.006 \| 0.003 \| -1.730 \| 0.084 \| 11,031 \| 113,141 \| \| **JILIN** \| Expen. (log) \| 0.843 \| 0.020 \| 41.568 \| 0.000 \| 4,104 \| 43,747 \| \| **JILIN** \| Mortality \| -0.007 \| 0.002 \| -4.416 \| 0.000 \| 4,104 \| 43,747 \| \| **HEILONGJIANG** \| Expen. (log) \| 0.563 \| 0.030 \| 19.014 \| 0.000 \| 14,854 \| 66,006 \| \| **HEILONGJIANG** \| Mortality \| -0.009 \| 0.004 \| -2.292 \| 0.022 \| 14,854 \| 66,006 \| \| **JIANGSU** \| Expen. (log) \| 0.532 \| 0.010 \| 55.231 \| 0.000 \| 26,992 \| 301,693 \| \| **JIANGSU** \| Mortality \| -0.001 \| 0.001 \| -1.049 \| 0.294 \| 26,992 \| 301,693 \| \| **ZHEJIANG** \| Expen. (log) \| 0.480 \| 0.071 \| 6.764 \| 0.000 \| 23,501 \| 146,971 \| \| **ZHEJIANG** \| Mortality \| -0.006 \| 0.003 \| -1.660 \| 0.097 \| 23,501 \| 146,971 \| \| **ANHUI** \| Expen. (log) \| 0.397 \| 0.019 \| 20.701 \| 0.000 \| 23,819 \| 100,418 \| \| **ANHUI** \| Mortality \| -0.005 \| 0.002 \| -2.511 \| 0.012 \| 23,819 \| 100,418 \| \| **FUJIAN** \| Expen. (log) \| 0.342 \| 0.040 \| 8.481 \| 0.000 \| 18,277 \| 97,579 \| \| **FUJIAN** \| Mortality \| -0.001 \| 0.002 \| -0.316 \| 0.752 \| 18,277 \| 97,579 \| \| **JIANGXI** \| Expen. (log) \| 0.347 \| 0.013 \| 27.431 \| 0.000 \| 34,229 \| 180,797 \| \| **JIANGXI** \| Mortality \| -0.005 \| 0.001 \| -6.335 \| 0.000 \| 34,229 \| 180,797 \| \| **SHANDONG** \| Expen. (log) \| 0.181 \| 0.024 \| 7.648 \| 0.000 \| 17,279 \| 134,411 \| \| **SHANDONG** \| Mortality \| -0.002 \| 0.002 \| -1.454 \| 0.146 \| 17,279 \| 134,411 \| \| **HENAN** \| Expen. (log) \| 0.454 \| 0.011 \| 40.096 \| 0.000 \| 87,054 \| 153,597 \| \| **HENAN** \| Mortality \| -0.002 \| 0.001 \| -2.844 \| 0.004 \| 87,054 \| 153,597 \| \| **HUBEI** \| Expen. (log) \| 0.535 \| 0.012 \| 43.465 \| 0.000 \| 46,869 \| 391,415 \| \| **HUBEI** \| Mortality \| -0.002 \| 0.001 \| -2.125 \| 0.034 \| 46,869 \| 391,415 \| \| **HUNAN** \| Expen. (log) \| 0.791 \| 0.026 \| 30.541 \| 0.000 \| 16,076 \| 53,438 \| \| **HUNAN** \| Mortality \| 0.000 \| 0.001 \| -0.380 \| 0.704 \| 16,076 \| 53,438 \| \| **GUANGDONG** \| Expen. (log) \| 0.289 \| 0.016 \| 18.491 \| 0.000 \| 87,213 \| 313,748 \| \| **GUANGDONG** \| Mortality \| -0.008 \| 0.001 \| -5.840 \| 0.000 \| 87,213 \| 313,748 \| \| **GUANGXI** \| Expen. (log) \| 0.267 \| 0.026 \| 10.385 \| 0.000 \| 24,933 \| 146,245 \| \| **GUANGXI** \| Mortality \| -0.003 \| 0.002 \| -1.365 \| 0.172 \| 24,933 \| 146,245 \| \| **HAINAN** \| Expen. (log) \| 0.209 \| 0.109 \| 1.923 \| 0.054 \| 5,361 \| 23,136 \| \| **HAINAN** \| Mortality \| 0.000 \| 0.007 \| -0.038 \| 0.970 \| 5,361 \| 23,136 \| \| **CHONGQING** \| Expen. (log) \| -0.025 \| 0.021 \| -1.208 \| 0.227 \| 37,404 \| 232,068 \| \| **CHONGQING** \| Mortality \| -0.002 \| 0.002 \| -1.145 \| 0.252 \| 37,404 \| 232,068 \| \| **SICHUAN** \| Expen. (log) \| 0.379 \| 0.076 \| 4.993 \| 0.000 \| 11,452 \| 55,990 \| \| **SICHUAN** \| Mortality \| 0.001 \| 0.004 \| 0.193 \| 0.847 \| 11,452 \| 55,990 \| \| **GUIZHOU** \| Expen. (log) \| 0.318 \| 0.015 \| 21.653 \| 0.000 \| 36,571 \| 174,649 \| \| **GUIZHOU** \| Mortality \| -0.005 \| 0.001 \| -3.231 \| 0.001 \| 36,571 \| 174,649 \| \| **SHAANXI** \| Expen. (log) \| 0.652 \| 0.050 \| 13.108 \| 0.000 \| 16,699 \| 72,741 \| \| **SHAANXI** \| Mortality \| -0.001 \| 0.004 \| -0.352 \| 0.725 \| 16,699 \| 72,741 \| \| **GANSU** \| Expen. (log) \| 0.456 \| 0.080 \| 5.718 \| 0.000 \| 950 \| 13,170 \| \| **GANSU** \| Mortality \| -0.001 \| 0.006 \| -0.216 \| 0.829 \| 950 \| 13,170 \| |
| --- | --- | --- | --- | --- | --- | --- | --- | --- | --- | --- | --- | --- | --- | --- | --- | --- | --- | --- | --- | --- | --- | --- | --- | --- | --- | --- | --- | --- | --- | --- | --- | --- | --- | --- | --- | --- | --- | --- | --- | --- | --- | --- | --- | --- | --- | --- | --- | --- | --- | --- | --- | --- | --- | --- | --- | --- | --- | --- | --- | --- | --- | --- | --- | --- | --- | --- | --- | --- | --- | --- | --- | --- | --- | --- | --- | --- | --- | --- | --- | --- | --- | --- | --- | --- | --- | --- | --- | --- | --- | --- | --- | --- | --- | --- | --- | --- | --- | --- | --- | --- | --- | --- | --- | --- | --- | --- | --- | --- | --- | --- | --- | --- | --- | --- | --- | --- | --- | --- | --- | --- | --- | --- | --- | --- | --- | --- | --- | --- | --- | --- | --- | --- | --- | --- | --- | --- | --- | --- | --- | --- | --- | --- | --- | --- | --- | --- | --- | --- | --- | --- | --- | --- | --- | --- | --- | --- | --- | --- | --- | --- | --- | --- | --- | --- | --- | --- | --- | --- | --- | --- | --- | --- | --- | --- | --- | --- | --- | --- | --- | --- | --- | --- | --- | --- | --- | --- | --- | --- | --- | --- | --- | --- | --- | --- | --- | --- | --- | --- | --- | --- | --- | --- | --- | --- | --- | --- | --- | --- | --- | --- | --- | --- | --- | --- | --- | --- | --- | --- | --- | --- | --- | --- | --- | --- | --- | --- | --- | --- | --- | --- | --- | --- | --- | --- | --- | --- | --- | --- | --- | --- | --- | --- | --- | --- | --- | --- | --- | --- | --- | --- | --- | --- | --- | --- | --- | --- | --- | --- | --- | --- | --- | --- | --- | --- | --- | --- | --- | --- | --- | --- | --- | --- | --- | --- | --- | --- | --- | --- | --- | --- | --- | --- | --- | --- | --- | --- | --- | --- | --- | --- | --- | --- | --- | --- | --- | --- | --- | --- | --- | --- | --- | --- | --- | --- | --- | --- | --- | --- | --- | --- | --- | --- | --- | --- | --- | --- | --- | --- | --- | --- | --- | --- | --- | --- | --- | --- | --- | --- | --- | --- | --- | --- | --- | --- | --- | --- | --- | --- | --- | --- | --- | --- | --- | --- | --- | --- | --- | --- | --- | --- | --- | --- | --- | --- | --- | --- | --- | --- | --- | --- | --- | --- | --- | --- | --- | --- | --- | --- | --- | --- | --- | --- | --- | --- | --- | --- |

Abbreviations: CKD, chronic kidney disease; UBMI, urban basic medical insurance; NRCMC, new rural co-operative medical care.

Note: Beijing, Shanghai, Tibet, Qinghai, Ningxia, and Xingjiang are excluded due to insufficient observations of medical migrants. Taiwan, Hong Kong, and Macau are excluded due to no observations.

**Table S11. Sensitivity Tests based on different cost of transportation and lodging**

| Medical cost per life saved (RMB) | Transportation cost per KM (RMB) | Lodging cost per day (RMB) | Total cost per life saved (RMB) | |
| --- | --- | --- | --- | --- |
|  | | | |  |
| Panel A: Baseline estimate | | | |  |
| 1002,288 | 0.5 | 100 | 1,379,788 | |
|  | | | |  |
| Panel B: Different cost of transportation | | | |  |
| 1,002,288 | 0.25 | 100 | 1,347,288 | |
| 1,002,288 | 0.75 | 100 | 1,412,288 | |
| 1,002,288 | 1 | 100 | 1,444,788 | |
| 1,002,288 | 1.5 | 100 | 1,509,788 | |
|  | | | |  |
| Panel C: Different cost of lodging | | | |  |
| 1,002,288 | 0.5 | 50 | 1,223,538 | |
| 1,002,288 | 0.5 | 75 | 1,301,663 | |
| 1,002,288 | 0.5 | 150 | 1,536,038 | |
| 1,002,288 | 0.5 | 200 | 1,692,288 | |
|  |  |  |  | |

Note: Panel A reports the baseline estimates of cost per life saved of medical migration; Panel B for estimates under different assumed cost of trans-provincial transportation; Panel C for estimates under different assumed cost of lodging. Across panels B and C, we adopt the sample average transportation distance of 312 KM and the sample average length of lodging of 7.5 days.

# Appendix B: Figures

**Figure S1. Distribution of estimated propensity scores for baseline sample after PSM estimation**


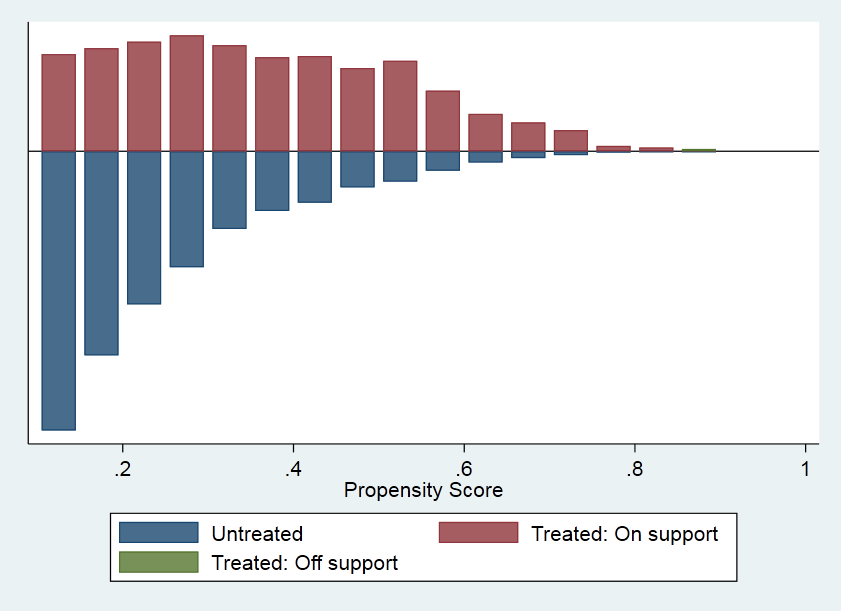


Abbreviations: PSM, propensity score matching.

Note: This figure plot the distribution of the estimated propensity scores for the baseline sample. Propensity score matching method is used to construct the matched sample of the treated group (migrant patients) and untreated group (non-migrant patients). The logistic model was used to estimate the propensity score based on the patient’s demographics, health insurance, major comorbidities, province dummies, and year dummies. Treated subjects were matched with the untreated subjects using nearest neighbor algorithm with a caliper of 0.1. The final sample was further restricted to have estimated scores strictly between 0.1 and 0.9. Off-support observations are also dropped.

**Figure S2. Distribution of estimated propensity scores for the sample with UBMI after PSM estimation**


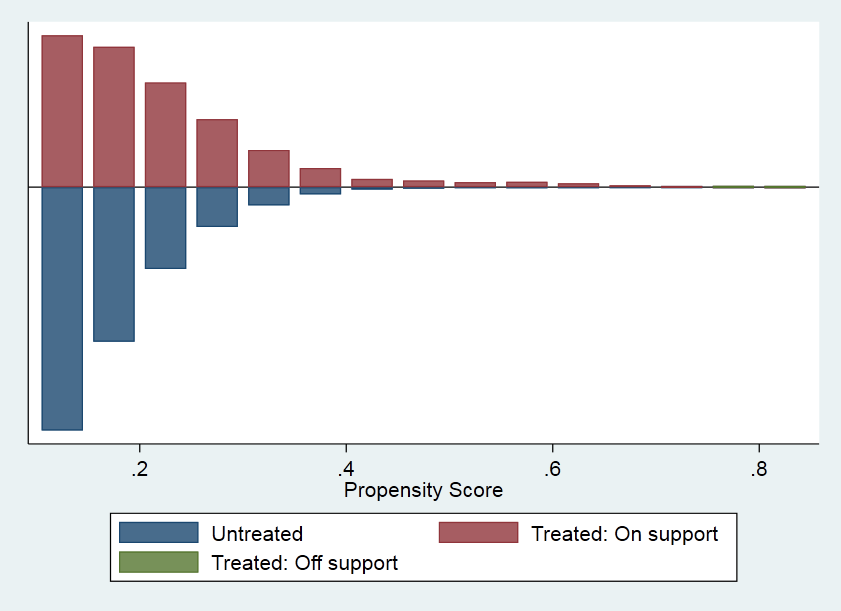


Abbreviations: PSM, propensity score matching; UBMI, urban basic medical insurance.

Note: This figure plot the distribution of the estimated propensity scores for the sample with UBMI insurance. Propensity score matching method is used to construct the matched sample of the treated group (migrant patients) and untreated group (non-migrant patients). The logistic model was used to estimate the propensity score based on the patient’s demographics, health insurance, major comorbidities, province dummies, and year dummies. Treated subjects were matched with the untreated subjects using nearest neighbor algorithm with a caliper of 0.1. The final sample was further restricted to have estimated scores strictly between 0.1 and 0.4. Off-support observations are also dropped.

**Figure S3. Distribution of estimated propensity scores for the sample with NRCMC after PSM estimation**


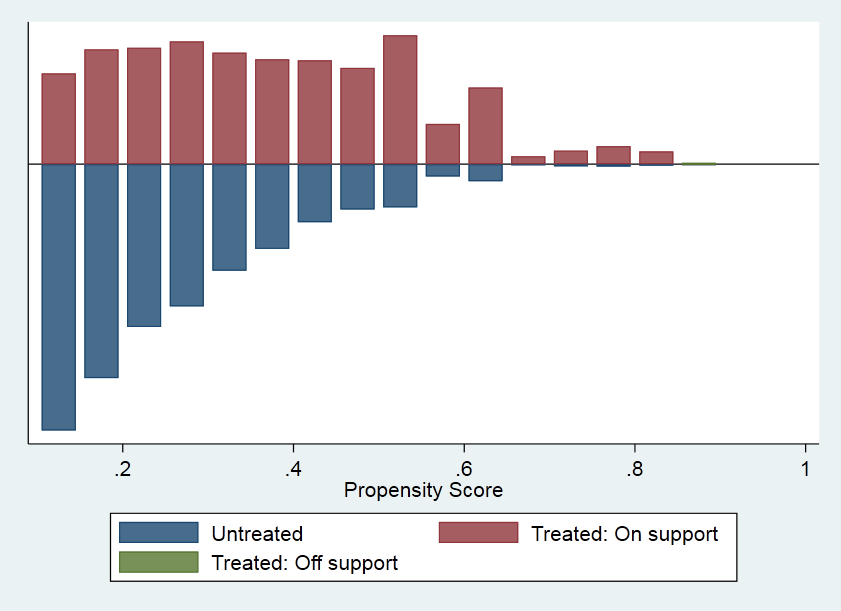


Abbreviations: PSM, propensity score matching; NRCMC, new rural co-operative medical care.

Note: This figure plot the distribution of the estimated propensity scores for the sample with NRCMC insurance. Propensity score matching method is used to construct the matched sample of the treated group (migrant patients) and untreated group (non-migrant patients). The logistic model was used to estimate the propensity score based on the patient’s demographics, health insurance, major comorbidities, province dummies, and year dummies. Treated subjects were matched with the untreated subjects using nearest neighbor algorithm with a caliper of 0.1. The final sample was further restricted to have estimated scores strictly between 0.1 and 0.8. Off-support observations are also dropped.

**Figure S4. Distribution of estimated propensity scores for the sample with self-payment after PSM estimation**


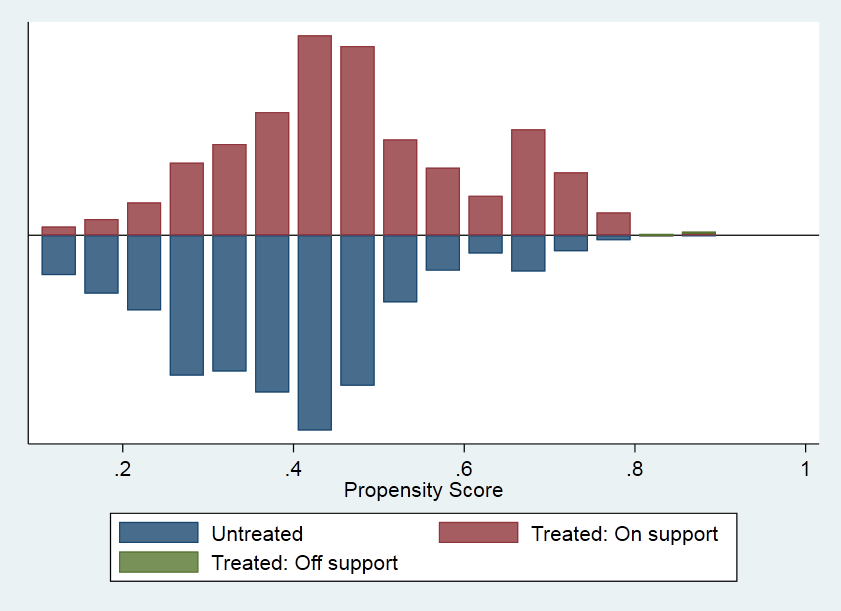


Abbreviations: PSM, propensity score matching.

Note: This figure plot the distribution of the estimated propensity scores for the sample with self payment. Propensity score matching method is used to construct the matched sample of the treated group (migrant patients) and untreated group (non-migrant patients). The logistic model was used to estimate the propensity score based on the patient’s demographics, health insurance, major comorbidities, province dummies, and year dummies. Treated subjects were matched with the untreated subjects using nearest neighbor algorithm with a caliper of 0.1. The final sample was further restricted to have estimated scores strictly between 0.1 and 0.8. Off-support observations are also dropped.

**Figure S5. Distribution of estimated propensity scores for the sample with other health insurance after PSM estimation**


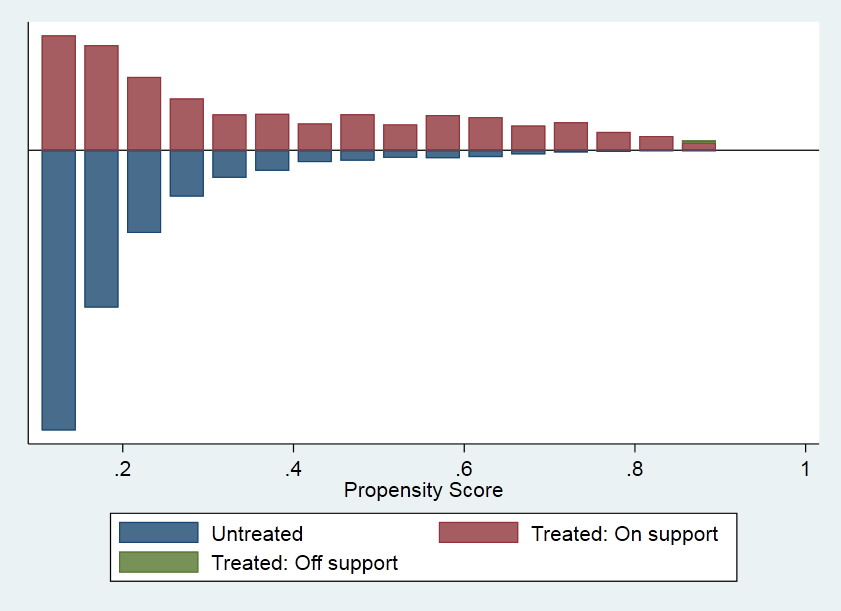


Abbreviations: PSM, propensity score matching.

Note: This figure plots the distribution of the estimated propensity scores for the sample with other health insurance, including commercial insurance and other types of health insurance. Propensity score matching method is used to construct the matched sample of the treated group (migrant patients) and untreated group (non-migrant patients). The logistic model was used to estimate the propensity score based on the patient’s demographics, health insurance, major comorbidities, province dummies, and year dummies. Treated subjects were matched with the untreated subjects using nearest neighbor algorithm with a caliper of 0.1. The final sample was further restricted to have estimated scores strictly between 0.1 and 0.8. Off-support observations are also dropped.
